# Barriers and facilitators for implementation of a combined lifestyle intervention in community-dwelling older adults: a scoping review

**DOI:** 10.3389/fpubh.2023.1253267

**Published:** 2023-10-11

**Authors:** Patricia J. van der Laag, Berber G. Dorhout, Aaron A. Heeren, Cindy Veenhof, Di-Janne J. A. Barten, Lisette Schoonhoven

**Affiliations:** ^1^Julius Center for Health Sciences and Primary Care, Nursing Science, University Medical Center Utrecht, University Utrecht, Utrecht, Netherlands; ^2^Research Group Innovation of Human Movement Care, Research Centre for Healthy and Sustainable Living, Utrecht University of Applied Sciences, Utrecht, Netherlands; ^3^Division of Human Nutrition and Health, Wageningen University and Research, Wageningen, Netherlands; ^4^Department of Rehabilitation, Physical Therapy Science & Sports, University Medical Center Utrecht, Utrecht University, Utrecht, Netherlands; ^5^Center for Physical Therapy Research and Innovation in Primary Care, Julius Health Care Centers, Utrecht, Netherlands; ^6^Faculty of Health Sciences, University of Southampton, Southampton, United Kingdom

**Keywords:** determinants, community, implementation, lifestyle intervention, older adults

## Abstract

**Background:**

Lifestyle interventions, combining nutrition and exercise, are effective in improving the physical functioning of community-dwelling older adults and preventing healthcare risks due to loss in muscle mass. However, the potential of these types of interventions is not being fully exploited due to insufficient implementation. Having insight into the determinants that could hinder or facilitate the implementation of a combined lifestyle intervention could improve the development of matching implementation strategies and enhance the implementation of such lifestyle interventions. The aim of this study was to identify barriers and facilitators for the successful implementation of a combined lifestyle intervention for community-dwelling older adults.

**Method:**

A scoping review was conducted. A literature search was conducted in four electronic databases, and references were checked for additional inclusion. Studies were screened if they met the inclusion criteria. Barriers and facilitators were extracted from the included studies. To validate the results of the literature search, healthcare professionals and community-dwelling older adults were interviewed. Barriers and facilitators were categorized by two researchers according to the constructs of the Consolidated Framework for Implementation Research (CFIR).

**Results:**

The search identified 12,364 studies, and 23 were found eligible for inclusion in the review. Barriers and facilitators for 26 of the 39 constructs of the CFIR were extracted. The interviews with healthcare professionals and older adults yielded six extra barriers and facilitators for implementation, resulting in determinants for 32 of the 39 CFIR constructs. According to literature and healthcare professionals, cosmopolitanism (network with external organizations), patient needs and resources, readiness for implementation, costs, knowledge and beliefs about the intervention, network and communication, and engaging were found to be the most important determinants for implementation of a combined lifestyle intervention.

**Conclusion:**

A broad range of barriers and facilitators across all domains of the CFIR framework emerged in this study. The results of this review reflect on determinants that should be taken into account when planning for the implementation of a combined lifestyle intervention. A further step in the implementation process is the development of implementation strategies aiming at the identified determinants to enhance the implementation of a combined lifestyle intervention in community care.

## 1. Introduction

The world's population is aging rapidly (1). Aging is associated with a decline in muscle mass and strength, which ultimately can lead to a decrease in physical function and quality of life (2–4). Decreased physical functioning is a predictor of disability and loss of independence. Older adults with mobility disabilities are found to have higher rates of hospital admissions, depression, morbidity, and even mortality (5).

Although physical decline is inevitable, a healthy lifestyle is found to delay this age-related deterioration of health. Exercise and physical activity, healthy weight, healthy nutrition, and participation in joyful activities are themes that are included in various guidelines of healthcare associations to encourage a healthy lifestyle in older adults (6, 7). Physical activity is an effective contributor to counteracting physical decline. However, a multifactorial approach combining physical activity with, for example, healthy nutrition, is more effective to obtain and maintain a healthy lifestyle (1). In most cases, attention to such lifestyle aspects is offered in so-called combined lifestyle interventions.

Combined lifestyle interventions targeting multiple aspects of obtaining and maintaining a healthy lifestyle showed promising results in several populations. In addition to widespread positive effects in people with chronic disabilities or illnesses (7–9), effective interventions are described in the general population of older adults in primary care (10, 11). One of these combined lifestyle interventions is ProMuscle. ProMuscle targets community-dwelling older adults (>65 years) and combines resistance exercise training with dietary protein intake (12–14). ProMuscle was found to be effective in improving muscle strength, lean body mass, and physical functioning in community-dwelling older adults (4, 14, 15).

Despite the promising effects of ProMuscle, it is not self-evident that ProMuscle is already widely used in daily practice. Unfortunately, this “evidence to practice gap” is not unique to ProMuscle. It reflects many of the hundreds of evidence-based programs that are thoroughly investigated and found effective every year. Just a small amount, 20% of the evidence-based programs, is actually implemented in daily practice (16), which, among others, results in a waste of research funding (17). Moreover, there is a chance that older adults do not receive new evidence-based care to prevent them from health-related complications, consequently leading to increasing healthcare costs (18). Therefore, successful implementation of evidence-based, combined lifestyle interventions such as ProMuscle is necessary, given the increasing population of older adults (19).

Implementation success is not only enhanced by an effective intervention, but a large number of contextual factors also play an important role in the perceived implementation success. Proctor et al. suggested an equation to model implementation success where it depends on the effectiveness of the treatment and the implementation factors (such as attitudes, behavior, and contextual factors) (20). Therefore, it is important to identify contextual factors so that implementation can be carefully planned, and strategies strategically employed. Moreover, implementation is a complex process (16) and comprises multiple steps to enhance the chance of successful implementation. To deal with this complexity, it is important to use a systematic approach and careful planning of the implementation process (21). Mapping the context in which the innovation is implemented, including identifying barriers and facilitators for implementation, is one essential step in this process.

It is known that a large number of determinants can hinder or facilitate successful implementation and may arise on several levels (21–23). Some studies already identified determinants that could hinder or facilitate the implementation of a combined lifestyle intervention. For example, Belizan et al. (24) identified financial resources, support and acceptance by local authorities and the community, and training as determinants that could influence the implementation of interventions that promote physical activity and a healthy diet. In addition, other studies investigated determinants for the implementation of interventions for people with specific health-related diagnoses (9, 25). However, the implementation of a combined lifestyle intervention for community-dwelling older adults is probably affected by other determinants. To date, no other research identified the determinants that could hinder or facilitate the implementation of a lifestyle intervention combining exercise for community-dwelling older adults.

Therefore, the aim of this study was to identify barriers and facilitators for the successful implementation of a combined lifestyle intervention for community-dwelling older adults in primary care.

## 2. Methods

A scoping review was conducted according to the framework for scoping reviews described by Arksey & O'Malley and the Joanna Briggs Institute guidelines (26, 27). The six steps of this framework describe a structured guidance to conduct a scoping review: (1) identifying the research question, (2) identifying relevant studies, (3) selecting studies, (4) charting the data, (5) collecting, summarizing, and reporting the results, and (6) consulting with key stakeholders. The method and analysis of these six steps are described below (26). The Preferred Reporting Items for Systematic Reviews and Meta-analyses Extension for Scoping Review (PRISMA-ScR) (28) was used to guide the reporting of the methodology and results. This study was approved by the Medical Committee University Utrecht (22/050). All participants gave written informed consent.

### 2.1. Research question

The research question of this scoping review is as follows: Which determinants hinder or facilitate the successful implementation of a combined lifestyle intervention for community-dwelling older adults in primary care?

### 2.2. Search strategy

A search strategy was designed in consultation with an experienced research librarian at Utrecht University. Four electronic databases with a scope in health(care) were searched: PubMed, Embase, CINAHL, and PsychInfo. In addition, reference lists of the included studies were screened for additional studies. Studies were screened on the following eligibility criteria: (1) Intervention was aimed at community-dwelling older adults (>65 years); (2) studies described a multicomponent intervention that contains at least an exercise component; (3) studies described perceived barriers and/or facilitators for implementation; (4) studies were published in English or Dutch; and (5) full texts were available. Published studies using quantitative, qualitative, or mixed-methods designs were considered eligible, as well as conference abstracts. Studies were excluded if (1) the intervention was delivered exclusively as an e-health or web-based intervention and (2) the intervention was aimed at a specific patient group (e.g., cancer, addiction, and mental health problems). Moreover, case studies and literature reviews were excluded. The extended search strategy for PubMed is presented in Appendix 1.

### 2.3. Study selection

First, all retrieved publications were uploaded in Rayyan (29) and duplicates were removed. Hereafter, two researchers screened titles and abstracts for eligible studies. Of the studies that were deemed eligible for further inclusion, two researchers (PL and AH) independently assessed the full-text publications to see whether they met the inclusion criteria. Studies that did not meet the criteria were excluded, and the reason for exclusion was listed. In case of disagreement between the two researchers, a discussion took place to reach consensus. If consensus was not reached, a third researcher (DB) was consulted.

### 2.4. Charting the data

A standardized data extraction form was developed before the data extraction. One researcher extracted data from the included studies. The following data were extracted: Author, country, study design, participants, intervention, and barriers and facilitators for implementation. Two reviewers (PL and AH) discussed the extracted data from all included studies to ensure the reliability of the extraction process.

### 2.5. Collating, summarizing, and reporting the results

Barriers and facilitators for successful implementation of a combined lifestyle intervention were identified from the results or discussion section of the included studies. Determinants were extracted through thematic analysis and deductively categorized into the constructs of the five main domains of the Consolidated Framework for Implementation Research (CFIR) by two researchers (PL and AH).

The original CFIR was used to categorize the identified barriers and facilitators (23). The CFIR describes implementation determinants from different implementation theories and is composed of five major domains (i.e., intervention characteristics, outer setting, inner setting, characteristics of individuals, and process) that are made up of 39 constructs that influence the implementation of innovations into practice (23). The CFIR domains with their constructs are presented in Appendix 2.

Discrepancies were discussed and, if necessary, consensus about the definitions of the CFIR constructs was reached to categorize identified determinants in the best matching CFIR construct.

### 2.6. Consultation of key stakeholders

To validate the results of the literature search and to prioritize the relevance of the identified determinants (30), consultation groups with relevant healthcare professionals and interviews with community-dwelling older adults were conducted.

#### 2.6.1. Consultation group with healthcare professionals

Four online consultation groups were conducted with a convenience sample of 13 physical therapists, 3 dieticians, and 2 lifestyle coaches who were interested in implementing the combined lifestyle intervention “ProMuscle.” Consultation group participants were recruited in the region of Utrecht and Gelderland (the Netherlands) through local networks and social media. Healthcare professionals were eligible to participate if they had experience in working with older adults (>65 years). A mixed group of healthcare professionals was included in the consultation groups since it was expected to lead to an additional discussion to explore the specific context and to ensure that the results are relevant for daily practice.

The consultation groups were led by an experienced researcher as moderator (BD). A second researcher (AH) observed, took notes, and supported the moderator when necessary.

A semi-structured interview guide was based on the barriers and facilitators identified from the literature and was structured in line with the five domains of the CFIR and corresponding constructs. To ensure a thorough exploration of barriers and facilitators, only two CFIR domains were discussed per consultation group. The CFIR domain “Intervention characteristics” was not discussed in the consultation groups, as participating healthcare professionals were not yet familiar with the combined lifestyle intervention “ProMuscle.” Each consultation group started with a short introduction to explain the definition of the CFIR domains and the constructs to be discussed. Participants were first asked to rank the identified determinants of relevance for implementation per domain. Hereafter, a guided discussion took place to operationalize the determinants by providing examples from their daily practice. Finally, participants were asked for missing determinants.

#### 2.6.2. Interviews with community-dwelling older adults

To understand specifically which barriers and facilitators community-dwelling older adults experience when they consider to participate in a combined lifestyle intervention, three older residents, two men, and one woman with a mean age of 70 years, living in Utrecht or Gelderland in the Netherlands were interviewed face-to-face in July 2021. These older residents were recruited by healthcare professionals who participated in the consultation group sessions. A semi-structured interview guide was developed based on the identified barriers and facilitators within the CFIR construct “patient needs.”

#### 2.6.3. Data analysis

The consultation groups and interviews were transcribed verbatim. Transcripts were coded following a deductive approach using the Framework method (31). First, two researchers (PL and BD) read the transcripts independently line-by-line to identify emerging concepts and coded the data (32) into the CFIR constructs. Data that did not fit in one of the CFIR constructs were coded as additional codes. The researchers eventually compared the coding and resolved discrepancies through discussion. Nvivo (version 12) was used to analyze transcripts.

## 3. Results

### 3.1. Study selection

**Figure 1:**
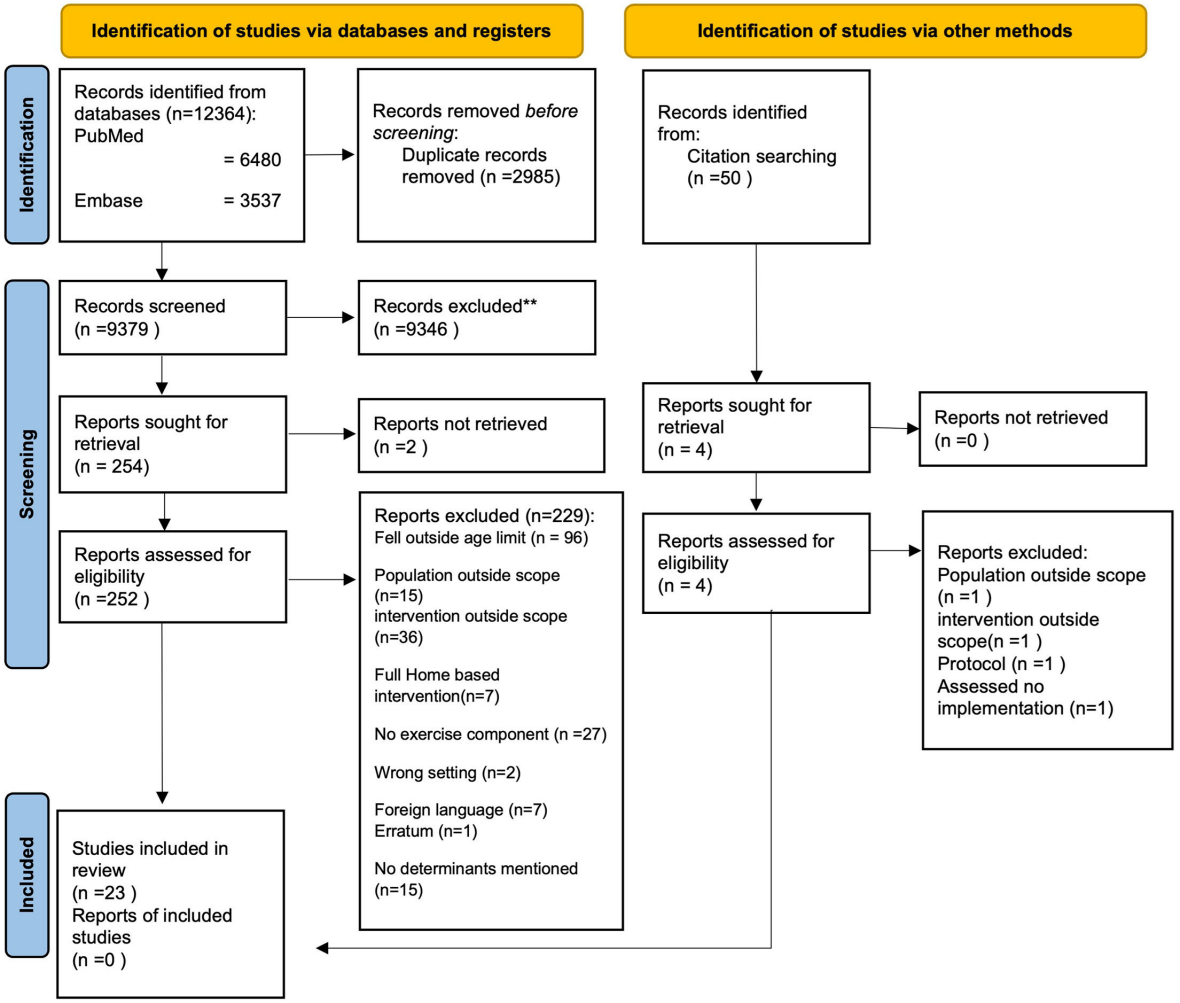
PRISMA flowchart. Identification of studies.

In total, 12,364 studies were identified in PubMed, Embase, CINAHL, and PsychInfo. After removing duplicates, the title and abstract of 9,379 studies were screened for eligibility. A total of 252 full-text articles were screened for further inclusion. Of the 252 studies, 229 studies were excluded. The main reasons for exclusion were as follows: Participants fell outside the age range of >65 years, implementation was conducted in a population outside the scope of the study, or studies implemented interventions outside the scope of the study, and studies did not assess the implementation of the intervention. No additional studies could be included through citation searching. Ultimately, 23 studies were included in this review (Figure 1).

### 3.2. Characteristics of the included studies

The characteristics of the 23 included studies are presented in Table 1.

**Table 1:** Study characteristics of the included studies.

**References**	**Country**	**Study design**	**Participants**	**Intervention**
Ayton et al. (33)	Australia	Mixed methods	Clients of personal alert Victoria	Fall prevention intervention
Banez et al. (34)	Canada	Quantitative non-randomized	Seniors	Fall prevention intervention
Baxter et al. (35)	Canada	Qualitative	Healthcare team members	Fall prevention intervention
Bobitt et al. (18)	USA	Qualitative	Directors of area agencies on aging (AAA) and senior center leader	Community-based EB lifestyle programs
Brown et al. (36)	USA	Qualitative (conference abstract)	Representatives of aging services organizations	Healthy aging and chronic disease prevention and management EBI
Corcoran et al. (5)	USA	Randomized control trial	Facility staff	Exercise and nutritional supplement program
van Dongen et al. (13)	The Netherlands	Mixed methods	Older community living adults and healthcare professionals	Dietary protein and exercise intervention
van Dongen et al. (4)	The Netherlands	Mixed methods	Community-dwelling older adults and healthcare professionals	Intensive support intervention (exercise and dietary protein)
Ford et al. (37)	USA	A cluster-randomized control group design.	Rural county aging unit staff	Implementation strategy for increasing uptake of 2 health promotion programs
Gavarkovs et al. (38)	Canada	Qualitative	Rural program delivery staff	Community-based chronic disease prevention and management program
Horning et al. (39)	USA	Quantitative non-randomized	Community-dwelling older adults	Brain training (psycho-education and exercise)
Hui-Chuan Hsu et al. (40)	Taiwan	Quantitative cohort	Older adults and trainers	Community-based aging intervention (physical activity and nutrition)
Hui-Chuan Hsu et al. (41)	Taiwan	Mixed methods	Unknown	Successful aging program (lecture about successful aging, nutrition, chronic disease, financial security, internet use + physical activity + cognitive function training)
Jyväkorpi et al. (2)	Europe	Quantitative non-randomized	Nutrition interventionists	Physical activity and nutrition intervention
Kulmala et al. (42)	Finland	Qualitative	Healthcare professionals and managers	Mental health intervention
Liddle et al. (43)	Unknown	Qualitative	Allied health professionals	Fall prevention intervention
Mackenzie et al. (44)	Australia, UK, Canada	Qualitative	Health professionals	Fall prevention intervention
Markle-Reid et al. (45)	Canada	Mixed Methods	Providers, peer support volunteers, receivers of diabetes-related services	Community-based program of diabetes self-management
Middlebrook et al. (46)	Australia	Qualitative	Private occupational therapists and physiotherapists	Enhanced primary care program
Paone et al. (47)	Corresponding: USA	Mixed methods	State representatives and organizational representatives	Chronic disease self-management program
Smith et al. (48)	USA	Quantitative	Recipients of funds	Fall prevention intervention
Taing et al. (49)	Canada	Mixed methods	Public health staff and recreation, cultural and facilities service staff, instructors, community-dwelling older adults	Fall prevention intervention
Zachary et al. (50)	USA	Cross-sectional Quantitative	Senior center directors and activities directors	Fall prevention intervention

EB = Evidence-based.

The included studies were published between 2008 and 2021. Seven studies had a qualitative design (18, 35, 38, 42–44, 46), eight a quantitative study design (2, 5, 34, 37, 39, 40, 48, 50), and seven a mixed-methods design (4, 13, 33, 41, 45, 47, 49). One study was a conference abstract (36). Seven studies were conducted in the USA (5, 18, 36, 37, 39, 48, 50), five in Canada (34, 35, 38, 45, 49), two in Taiwan (40, 41), two in Australia (33, 46), two in the Netherlands (4, 13), and one in Finland (42). One multicenter non-randomized study was conducted in Europe (2). One multicenter study was conducted in Canada, UK, and Australia (44). Two studies did not describe the location of their study, the corresponding author of one study was from the USA (47), and for the other study, it was impossible to determine the country in which the study was conducted.

The content of the interventions differed per study. Eight studies evaluated an integrated fall prevention intervention (33–35, 43, 44, 48–50), five studies a nutrition and exercise program (2, 4, 5, 13, 40), four studies evaluated a chronic disease self-management/prevention community-based program (36, 38, 45, 47), one study a successful aging program (41), one study a community-based evidence-based lifestyle program (18), one study a brain training (psycho-education and exercise) (39), one study a health promotion program (37), one study an enhanced primary care program (46), and one study a mental health program (42).

### 3.3. Barriers and facilitators for the implementation of combined lifestyle interventions in primary care

The studies in this literature review described barriers and facilitators as determinants for the implementation of a combined lifestyle intervention.

In sum, a total of 654 determinants were identified from the literature. The CFIR framework comprises a wide range of contextual factors that could influence implementation. The identified determinants that could influence the implementation of a combined lifestyle intervention for community-dwelling older adults were organized in the constructs of the five CFIR domains. For the 39 constructs of the CFIR framework, determinants influencing the implementation of a combined lifestyle intervention emerged for 32 constructs. An extra six determinants of the CFIR framework were identified from the consultation with healthcare professionals; *tension for change* (implementation climate), *organizational incentives and rewards* (implementation climate)*, relative advantage, peer pressure, self-efficacy*, and *external change agents*. Three determinants emerged from the literature but were not recognized by healthcare professionals (i.e., *Relative priority* (implementation climate), *individual stage of change*, and *reflecting and evaluating*). Determinants that older adults mentioned during the interviews were categorized and described in the domain outer setting (construct *Patients needs & resources*).

Table 2 presents the identified barriers and facilitators from the included studies categorized per CFIR domain and indicates if determinants were mentioned by healthcare professionals including the prioritization during the consultation groups.

**Table 2:** Barriers and facilitators identified from the included studies and consultation groups with healthcare professionals, categorized per CFIR construct including prioritization from healthcare professionals.

**Construct**	**Sub-component**	**Barrier/facilitator from literature**	**References**	**Emerged from stakeholder consultation?**	**Ranking of priority by HCP per construct per domain**
**Intervention characteristics**					
Intervention source		No barriers and facilitators		No	
Evidence strength and quality		-		Yes	Intervention characteristics were not prioritized by HCP
		+	(2, 4, 34, 37, 47, 49)		
		+/–			
Relative advantage		No barriers and facilitators		Yes	
Adaptability		-	(18, 36, 42)	Yes	
		+	(2, 4, 46)		
		+/–	(13, 45)		
Trialability		No barriers and facilitators		No	
Complexity		-	(18, 43, 45)	Yes	
		+	(47)		
		+/–			
Design quality and packaging		-	(45, 49)	Yes	
		+	(13, 42)		
		+/–	(4)		
Cost		-	(4, 13, 33, 36, 38, 41, 43, 46, 47, 49)	Yes	
		+	(13, 48)		
		+/–	(18, 40)		
**Outer setting**					
Patient needs and resources		-	(2, 18, 36, 43, 46, 47)	Yes	1
		+	(38, 45)		
		+/–	(4, 13, 33, 34, 40–42, 44)		
Cosmopolitanism		-	(43, 44)	Yes	2
		+	(4, 13, 18, 40, 42, 47–50)		
		+/–	(46)		
Peer pressure		No barriers and facilitators		Yes	
External policy and incentives		-	(5, 36, 43)	Yes	3
		+	(18, 37, 47–49)		
		+/–	(42)		
**Inner setting**					
Structural characteristics		-	(5)	Yes	
		+	(48)		
		+/–			
Networks and communications		-	(43, 44)	Yes	1
		+	(4, 13, 46, 47)		
		+/–	(35, 49)		
Culture		No barriers and facilitators		No	
Implementation climate		-	(5, 42)	Yes	3
		+	(35)		
		+/–			
	Tension for change	No barriers and facilitators		Yes	
	Compatibility	-	(43)	Yes	
		+	(2, 4, 45, 47)		
		+/–	(13, 18)		
	Relative priority	-	(18, 42, 43)	No	
		+			
		+/–			
	Organizational incentives & rewards	-		No	
		+	(50)		
		+/–			
	Goals and feedback	No barriers and facilitators		No	
	Learning climate	No barriers and facilitators		No	
Readiness for implementation		-	(5)	Yes	2
		+			
		+/–			
	Leadership engagement	-	(50)	Yes	
		+	(47)		
		+/–			
	Available resources	-	(5, 24, 34, 40, 46, 49, 50)	Yes	
		+	(4, 18, 47, 48)		
		+/–	(41, 42, 45)		
	Access to knowledge & information	-	(18)	Yes	
		+	(4, 5, 13, 37, 47)		
		+/–			
**Characteristics of individuals**					
Knowledge and beliefs about the intervention		-	(5, 18, 42, 50)	Yes	1
		+	(13, 45)		
		+/–			
Self-efficacy		No barriers and facilitators		Yes	
Individual stage of change		-	(5, 18)	No	
		+	(4, 13)		
		+/–			
Individual identification with organization		No barriers and facilitators		No	
Other personal attributes		-	(35, 40, 46, 47)	Yes	2
		+	(4, 13, 39)		
		+/–	(42, 45)		
**Process**					
Planning		-		Yes	2
		+	(13)		
		+/–			
Engaging		-	(18)	Yes	1
		+			
		+/–			
	Opinion leaders	-		Yes	
		+	(5)		
		+/–			
	Formally appointed internal implementation leaders	-		Yes	
		+	(42, 50)		
		+/–			
	Champions	-		Yes	
		+	(47)		
		+/–			
	External change agents	No barriers and facilitators		Yes	
	Key stakeholders	-	(34, 36, 46, 47)	No	
		+	(45)		
		+/–			
	Innovation participants	-	(47)	Yes	
		+	(13, 45)		
		+/–	(40)		
Executing		No barriers and facilitators		No	
Reflecting & evaluating		-			
		+	(4, 13, 37)	No	
		+/–			

Barriers and facilitators identified from literature are presented as; += facilitator, −= barrier, +/– = barrier and facilitator.

For all five CFIR domains, the determinants categorized in the constructs of the CFIR will be described per CFIR domain below.

#### 3.3.1. Intervention characteristics

In the domain intervention characteristics, *costs* and *design quality and packaging* were determinants that were described in most literature. Although not specifically asked during the consultation groups, healthcare professionals suggested that cost could be the most important determinant. In the literature search, 14 studies were included that described *cost* as a determinant for successful implementation, and five studies described *design quality and packaging* as a determinant that could influence the implementation of a combined lifestyle intervention. *Adaptability* and *evidence strength and quality* were identified in seven and six studies, respectively. Other determinants that emerged from the literature search were *relative advantage* and *complexity*. The reason that costs were explicitly mentioned by healthcare professionals is probably because most of the preventive lifestyle interventions are currently not covered by healthcare insurance in the Netherlands. This requires a lot of creativity from healthcare professionals to cover the costs of such an intervention.

“I *don't want the implementation to be dependent on money, this happens a lot. Lately I was at a meeting about sports where we design some initiatives. When some ideas emerged, some people yell can we afford that? I believe that a lot of ideas are being excluded because of that, and that is a shame*.”

Moreover, costs affect the possibility to implement a combined lifestyle intervention in healthcare practice in many ways. For example, a program has to be cost-effective to be able to sustain in daily healthcare. Another example healthcare professionals mentioned was that costs can affect the motivation of older adults to engage in a program. On the one hand, if the price is too high for something that older adults think they do not need yet, it scares them off. On the other hand, healthcare professionals believe that older adults should pay a small contribution to regain some intrinsic motivation to complete the program.

In addition, healthcare professionals mentioned that people with low socioeconomic status probably benefit most from a lifestyle intervention, but have little to no financial resources to contribute.

“*I notice that people who are less motivated drop out eventually. So you have to ask yourself, do we need to change everybody? And does everything have to be covered by the healthcare insurance?” “On the other hand, it could be a good thing if the program is covered by the health care insurance or other funding. Because, if you see which group of people have an insufficient intake and are less physically active and have a lot of comorbidities, most of the time they are people with a low social economic status. So yeah.”*

#### 3.3.2. Outer setting

In the domain outer setting, the determinants *Cosmopolitanism (*working with external stakeholders*)* and *patient needs and resources* emerged in most included studies and were ranked as most important determinants by healthcare professionals.

Of the included studies, 16 studies described determinants in the domain outer setting, specifically for the construct “patient needs and resources.” Twelve studies described cosmopolitanism as a determinant for successful implementation.

Having the ability to work together with other healthcare professionals outside of the practice is important for the implementation of a combined lifestyle intervention. Moreover, according to the healthcare professionals, the general practitioners can have a major part in referring older adults to the intervention. To make that work, general practitioners should be aware and convinced of the benefits of the intervention.

“*We work in a health care center. So there are a lot of general practitioners in this building. Here we collaborate a lot and there are many referrals back and forth.”*

“*I think there is some kind of hierarchy, older people believe the general practitioner is the boss and take advice from him/her [general practitioner] more seriously. If a nurse practitioner or healthcare professional tells older people that they have discussed it with the general practitioner, they have more influence on peoples' motivation.”*

In addition, nine included studies described *external policies and incentives* as a determinant for successful implementation. In the consultation groups, healthcare professionals mentioned that a strong collaboration in the community between healthcare professionals and social workers could facilitate the implementation, but also contact with government and healthcare insurers could facilitate the implementation. For example, to explore funding possibilities. However, healthcare professionals also mentioned that it takes a lot of time to make contact, convince the local government about the success of an intervention, and provide the funders with results. The degree of networking also depends on an organization's vision. Some healthcare professionals mentioned that their organization does not prioritize external collaboration.

“*It is important that healthcare insurers are involved. You always have to prove that the program generates money or that it improves healthcare. But it cannot cost more money. But I believe that you can manage that a little bit, I am convinced of that!”*

#### 3.3.3. Perspectives of older adults participation in a combined lifestyle intervention

Community-dwelling older adults gave insight into reasons why they would or would not participate in a combined lifestyle intervention. Emerging determinants were categorized in the construct *patient needs and resources*. Most of the barriers and facilitators addressed by community-dwelling older adults are related to the motivation to participate in a lifestyle program. The content of the intervention is a major reason to participate in a program. Older adults mentioned that the social aspect of a program is a facilitator to participate, but that strict rules are a barrier to participate.

“*It is certainly fun. All are people who have similar difficulties.”*

“*If there are too many rules, in short, never mind…For example, if I receive a scheme what says when I have to eat what, it's just going too far.”*

Moreover, older people admitted that it is hard to see the benefits of consultations concerning protein-enriched nutrition. In contrast, seeing results and feeling you get healthier is important to people when it comes to the intervention.

“*I am a bit skeptical about the protein intake. I think it is a bit theoretical. Today you read in the newspaper about protein, the next day about carbs. And the next day, who knows, about something else. It is conflicting, in my opinion. Then I think, just let me do it my way…”*

“*One of the best parts was a rowing machine, where you saw the seconds counting down. And then, yeah, you can see what you are doing.”*

Older adults also indicated how much they were willing to pay for the program and how long they wanted to travel. Based on their indications, the mean amount of the costs for the intervention was approximately 50 euros per month. Travel time varied from 10 min by bike to 40 kilometers by car.

Finally, older adults indicated that ways to stimulate and involve people in the program were via email, advertising in a local newspaper, and community centers. A flyer could also work; however, it was mentioned that a flyer needs to be compact and attractive to the eye. In addition, older adults mentioned that the physical therapist or nurse practitioner could also refer people to the program.

“*Actually you have to see what it [the intervention] is about in a glimpse.”*

#### 3.3.4. Inner setting

From the literature search, *readiness for implementation (n* = *15)* and *network and communication* (*n* = 8) emerged in most included studies and were ranked to be the most important determinants by healthcare professionals within the domain inner setting. Available resources, a sub-construct of *readiness for implementation*, emerged in most included studies. Having access to knowledge and information, being provided by the organization, and having the available resources to implement a new intervention, for example, time, were facilitators for the implementation according to healthcare professionals. Moreover, having sufficient support within the organization is mentioned as an important determinant for successful implementation. The healthcare professionals indicated that prevention in healthcare for older adults is very important. However, some healthcare professionals doubt if prevention is one of the priorities of their organization, which could affect a successful implementation.

“*If there is insufficient support, you can do whatever you want, but it is flogging a dead horse.”*

Other determinants that emerged from the included studies were *implementation climate, leadership engagement, structural characteristics*, and *access to knowledge and information*.

#### 3.3.5. Characteristics of individuals

Constructs that emerged in most literature and during the consultation groups, in the domain characteristics of individuals, were *knowledge and beliefs about the intervention* and *other personal attributes*. A total of six studies described *knowledge and beliefs about the intervention* as a determinant for successful implementation, and nine studies described *other personal attributes* as a determinant. Healthcare professionals mentioned being enthusiastic, having the motivation to implement a new intervention, and type of employment were important personal attributes for a successful implementation.

“*The intention of a healthcare professional is important; you don't want to see it as a business model, but you have to believe in the importance of preventive care.”*

*Knowledge and beliefs* about the intervention was ranked as the most important determinant for implementation by healthcare professionals. Healthcare professionals mentioned that belief in the intervention is important to gain support within and outside the organization. To be able to inspire colleagues and to motivate older adults to start and complete the program are also important for healthcare professionals to believe in the intervention.

Besides having the knowledge and beliefs, healthcare professionals stated that people also have to show initiative in trying to implement the intervention. Other healthcare professionals added that having sufficient support from their organization remains necessary.

“*When you are an owner of a practice it is easier to convince people of your new idea.”*

“*You have to address it positive, not being too persistent just be super enthusiast. Taking initiative to join forces, you don't have to do it alone.”*

Four studies described *individual stage of change* as a determinant for successful implementation, but this determinant was not mentioned by healthcare professionals.

#### 3.3.6. Process

The determinant *engaging* within the domain process emerged most frequently in the literature as well as during the consultation group with healthcare professionals. To successfully implement a combined lifestyle intervention, having people who can motivate and stimulate people to change their work process is important. However, being able as a healthcare professional to attract and attach older adults to participate in the program is also important.

The construct *formally appointed internal implementation leaders*, a sub-construct of the construct *engaging*, was ranked as the most important determinant for implementation within the domain process by healthcare professionals.

To engage older adults in the intervention, multiple facilitators and barriers were discussed by healthcare professionals. As mentioned earlier, people have to be aware of the usefulness of the intervention, but professionals should also pay attention to the needs of older adults. Healthcare professionals believe that people should be informed about the importance of the program by healthcare professionals and general practitioners.

To recruit people for participation in a combined lifestyle intervention, multiple sources can be used, such as flyers, word of mouth, and advertising in local newspapers.

In addition, to keep older adults motivated, healthcare professionals should set up the intervention in a creative and patient-centered manner.

“*I think I stimulate and motivate older people verbally as well as during the therapy sessions. In that way, you make the therapy challenging for them and, yeah, include a bit of creativity.”*

In addition to the construct *engaging*, no other determinants emerged from consultation with healthcare professionals. *Planning* and *executing* were determinants described by one and three studies, respectively.

**Figure 2:**
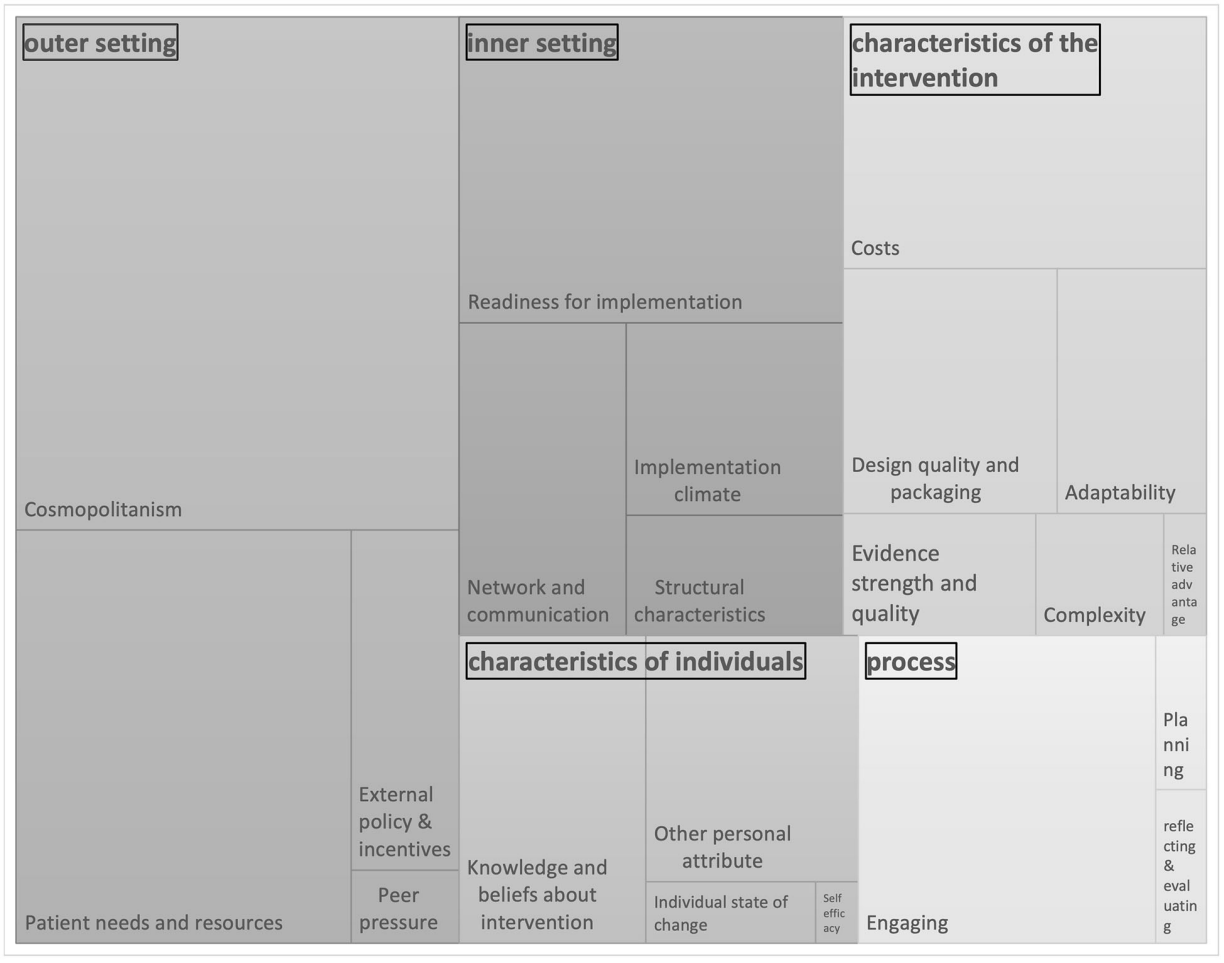
Barriers and facilitator for implementing a combined lifestyle intervention. Identified from literature and consultationgroups. The size of the box depends on the frequency of emergence of each CFIR construct.

In Figure 2, the determinants for the implementation of a combined lifestyle intervention from the literature and confirmed and prioritized by key stakeholders are visualized. All identified determinants clustered per CFIR domain are presented. The size of each box gives an indication of the frequency of emergence in literature in combination with the relevance according to stakeholders of each CFIR construct. For the domains intervention characteristics, outer setting, inner setting, characteristics of individuals, and process, the most frequently emerged constructs were *costs, cosmopolitanism, readiness for implementation, knowledge and beliefs about the intervention*, and *engaging*, respectively.

## 4. Discussion

This scoping review identified 654 different barriers and facilitators for the implementation of a combined lifestyle intervention for community-dwelling older adults.

The identified barriers and facilitators cover a broad range of determinants across all domains of the CFIR framework. The five constructs that most frequently emerged from literature and were confirmed by healthcare professionals are *cosmopolitanism* (network with external stakeholders, outer setting), *patient needs & resources* (outer setting), *readiness for implementation* (inner setting), *costs* (innovation characteristics), and *knowledge & beliefs about the intervention* (characteristics of individuals). In addition to the five most frequently emerged constructs, healthcare professionals ranked *network & communication* (inner setting) and *engaging* (process) to be the most relevant determinants for implementation. The determinants indicated by community-dwelling older adults mostly fit in the construct *patient needs and recourses*, whereas costs of the intervention was categorized in the construct *costs* (intervention characteristics). Older adults indicated that engaging and motivating, costs, and transportation to the intervention are determinants that could influence whether they participate in a combined lifestyle intervention or not.

Most identified determinants were categorized in the CFIR domain “outer setting.” This can be explained by the multicomponent character of the intervention. With a combined lifestyle intervention in older adults, multiple healthcare professionals are involved in the execution of the intervention. In general, few healthcare professionals work directly together and do not always have a constructive collaboration in the delivery of healthcare. Therefore, first, it is important to develop a collaboration between healthcare professionals to deliver the intervention correctly (51). In addition, to refer community-dwelling older adults to a combined lifestyle intervention, the general practitioner has a major role (9). Healthcare professionals mentioned that older adults would rather follow advice from a general practitioner than from family or a physical therapist. This is comparable with the study by Geense et al. (52), where it is suggested to let general practitioners ask older adults to participate in a lifestyle intervention because of the natural authority (52). However, other studies describe that GPs often do not have sufficient time to inform older adults properly. Moreover, without proper information, the quality of the advice can be affected (9). Similar to the study of Molema et al. (9), healthcare professionals in this review stated that not all general practitioners have a positive attitude toward lifestyle interventions. Therefore, a constructive collaboration with a general practitioner is necessary not only to refer eligible participants but also to inform and convince general practitioners about the benefits of the program so they can correctly pass the information to older adults.

Furthermore, the costs of the intervention emerged as an important determinant for implementation from the majority of the included studies and from the interviews with healthcare professionals and community-dwelling older adults. Not all lifestyle interventions, especially preventive lifestyle interventions, are structurally reimbursed by healthcare insurance in the Netherlands. This means that participants need to (partly) contribute to participate in the intervention. However, only a small percentage of older adults are willing to contribute every month to preventive healthcare. Moreover, not all older adults have the resources to pay for a lifestyle intervention. As a result, the majority of older adults will not benefit from proper preventive care, which ultimately can result in an increased risk of loss of independence and other healthcare-related risks (18). Consequently, the health differences will be preserved and probably increase. Finally, because of the temporality of most reimbursements and lack of structural funding, there is a great chance that a lifestyle intervention stops when funding ends (9).

Overall, the results of this study are in line with the small number of existing reviews reporting barriers and facilitators for combined lifestyle interventions. Belizan et al. (24) found that limited financial resources, lack of support and acceptance by the community, and lack of training were barriers to the implementation of health promotion interventions. In contrast, interventions with sufficient financial resources and support from local authorities and community members had more chance for successful implementation and sustainment of initiatives. This suggests, as also mentioned by the healthcare professionals in the current study, that it is important to have a constructive collaboration in the community with different partners to, for example, explore reimbursement possibilities for the intervention and the implementation process. In addition, studies that evaluated the implementation of combined lifestyle interventions for specific patient groups reported similar results as this study. For example, non-optimal interdisciplinary collaboration, negative attitudes toward the intervention, low literacy of patients, and lack of knowledge from HCP were barriers to the implementation of a combined lifestyle intervention for people with osteoarthritis (25). Moreover, structural funding, good infrastructure, and communication with stakeholders and motivated healthcare professionals were facilitators for the implementation of a combined lifestyle intervention for chronically ill people (9). Lack of time and costs were described as barriers. These barriers and facilitators could be categorized in the most frequently emerged CFIR constructs in this review: *cosmopolitanism, knowledge and beliefs about the intervention, patient needs and resources*, and *costs*. The similarities with the results of this review and other research suggest that the identified determinants in this scoping review are applicable for the implementation of an exercise and nutrition intervention such as ProMuscle as well as for other combined lifestyle interventions for older adults in general.

### 4.1. Strengths and limitations

A strength of this study was that it provides a first overview of the most common determinants in the great pile of possible contextual factors that could influence the implementation of a combined lifestyle intervention. Because of the qualitative explorative design, the validation of the results from the literature by stakeholders strengthens the findings of this study and ensures that the identified determinants influencing implementation fit the context in which the combined lifestyle intervention will be implemented. Another strength of this scoping review is that framework analysis was used to code the data, and three researchers analyzed it to prevent tunnel vision and to prevent new determinants from not being identified. This is amplified by the six extra constructs that were identified from the consultation group with stakeholders. The opposite was true for the determinants *relative priority, individual stage of change*, and *reflecting and evaluating*. These determinants were found in the literature but were not mentioned by healthcare professionals. Therefore, the question arises why six extra determinants yielded from the consultation group and another three determinants were not mentioned by healthcare professionals. An explanation could be that some determinants were not coded as the included studies intended. Moreover, in this research, the healthcare professionals were unfamiliar with the intervention and had no experience in implementing a combined lifestyle intervention. This is in contrast with the included studies, where a process evaluation was performed after the implementation of an intervention.

Because healthcare professionals were not experienced in the implementation of a combined lifestyle intervention, they were not questioned about the barriers and facilitators within the domain intervention characteristics. However, some of the identified determinants eventually could be categorized in the domain intervention characteristics as well. Most determinants reflected the general specifications of a combined lifestyle intervention, such as the costs of a preventive intervention (which is almost never reimbursed by healthcare insurance or local government in the Netherlands) or concerning the content of an intervention.

Although this review gives a first overview of determinants that could influence implementation, it was not possible to assess the weight of influence each determinant had on the implementation. This can be seen as a limitation of this study. However, this overview provides guidance in planning for implementation and development of implementation strategies. Another limitation of this study was that articles were excluded that did not describe barriers and facilitators for implementation in their abstracts. Many studies reflect on determinants that influenced the implementation of a combined lifestyle intervention during the trial. However, because the identification of barriers and facilitators is often not the aim of the studies, determinants are often not described in the abstract but mostly are mentioned in the discussion. This could have resulted in the exclusion of studies that did describe determinants for implementation. Despite the possibility that some studies were not included in the analysis, the results from this review correspond to other studies investigating barriers and facilitators for implementation. Therefore, we conclude that the results of this scoping review reflect the contexts where combined lifestyle interventions are implemented.

Finally, a limitation of this study was that only three older adults were interviewed to validate and identify determinants for participation in a combined lifestyle intervention. No distinct saturation was reached; however, most determinants were mentioned by all three older adults. In addition, five of the included studies described determinants from the recipients' perspective. The emerged determinants of this study could, except for one, be categorized in the construct *patient needs*. Furthermore, the identified determinants within the construct *Patient needs and resources* are similar to the results of other studies. For example, Herrema et al. (53) explored the drivers of compliance of older adults to a nutrition and exercise intervention. Support of a physical therapist, social contact, and knowledge about the benefits of the program motivated older adults to continue the program. At last, barriers were the high costs and the lack of an appropriate location. Location is not specifically mentioned by older adults in this scoping review. However, older adults mentioned that the transportation and the content of the intervention must be appealing to start with a lifestyle intervention. Therefore, the results of this scoping review concerning the construct *patient needs and resources* seem to provide an accurate impression of determinants to participate in a combined lifestyle intervention. Healthcare professionals and researchers can take these determinants into account when planning for the development and implementation of a combined lifestyle intervention.

The results of this review indicate that there are multiple determinants that could influence the implementation of a combined lifestyle intervention for older adults. As described in several implementation theories, the determinants found in this review also reflect on multiple levels in the setting where an intervention is implemented (21, 54). Moreover, it is expected that the influencing determinants for implementation are not similar for every setting (55). This review gives an overview of the most common determinants that could influence the implementation of a combined lifestyle intervention for community-dwelling older adults. When planning for the implementation of a combined lifestyle intervention, it is suggested to prioritize the determinants for the specific context to develop appropriate implementation strategies. In addition, the development of implementation strategies to enhance the implementation should focus on different levels and be evaluated to investigate whether strategies were suitable and effective to tackle the identified barriers for the implementation of a combined lifestyle intervention (56).

### 4.2. Conclusion

This scoping review identified 654 barriers and facilitators for the implementation of a combined lifestyle intervention for community-dwelling older adults. The barriers and facilitators are categorized into the five domains of the CFIR framework. The most frequently emerged determinants influencing implementation are as follows: *cosmopolitanism* (networking with external stakeholders, outer setting), *patient needs and resources* (outer setting), *readiness for implementation* (inner setting), *costs* (innovation characteristics), and *knowledge and beliefs about the intervention* (characteristics of individuals). A further step in the implementation process is the development of implementation strategies aiming at the identified determinants to enhance the implementation of a combined lifestyle intervention in primary care.

## Author contributions

BD, AH, and PL collected and analyzed the data from interviews with relevant stakeholders. AH, D-JB, and PL screened included articles for the study. AH and PL analyzed the data from the included studies. PL conducted the literature search, analyzed the qualitative and quantitative data, and wrote the manuscript. BD, AH, D-JB, CV, and LS provided feedback and input for the analysis of the data and critically read and provided feedback for the manuscript. All authors have read and approved the manuscript for submission.
