# Supplementary material for: Barriers and facilitators for implementation of a combined lifestyle intervention in community-dwelling older adults: a scoping review
Source: Front Public Health. 2023 Oct 11;11:1253267. doi: 10.3389/fpubh.2023.1253267 (PMC10602891; doi:10.3389/fpubh.2023.1253267)
Supplement: Supplementary file 1 [file Table_1.DOCX]

Supplementary Material

Barriers and facilitators for implementation of a combined lifestyle intervention in community-dwelling older adults: A scoping review

**Patricia J van der Laag*^1^, Berber G Dorhout^2,3^, Aaron A Heeren^2^, Cindy Veenhof^2,4,5^, Di-Janne JA Barten^2,4^, Lisette Schoonhoven^1,6^**

*** Correspondence:** Patricia J. van der Laag: p.j.vanderlaag-3@umcutrecht.nl

**Appendix 1. Search strategy PubMed**

 ("old"[Title/Abstract] OR "older adult*"[Title/Abstract] OR "elderly"[Title/Abstract] OR "aging"[Title/Abstract] OR "community-dwelling*"[Title/Abstract] OR "aged"[MeSH Terms] OR "aged, 80 and over"[MeSH Terms] OR "aged"[Title/Abstract] OR "frail elderly"[MeSH Terms] OR "frail elderly"[Title/Abstract] OR "geriatric patient*"[Title/Abstract] OR "frail patient*"[Title/Abstract] OR "older patient*"[Title/Abstract] OR "older people"[Title/Abstract] OR elder*[tiab] OR eldest[tiab] OR frail*[tiab] OR geriatri*[tiab] OR “old age*”[tiab] OR “oldest old*”[tiab] OR senior*[tiab] OR senium[tiab] OR “very old*”[tiab] OR septuagenarian*[tiab] OR octagenarian*[tiab] OR octogenarian*[tiab] OR nonagenarian*[tiab] OR “older people”[tiab] OR “older subject*”[tiab] OR “older age*”[tiab] OR “older adult*”[tiab] )

NOT ("Adolescent"[Mesh] OR "Child"[Mesh] OR "Infant"[Mesh] OR adolescen*[tiab] OR child*[tiab] OR schoolchild*[tiab] OR infant*[tiab] OR girl*[tiab] OR boy*[tiab] OR teen[tiab] OR teens[tiab] OR teenager*[tiab] OR youth*[tiab] OR pediatr*[tiab] OR paediatr*[tiab] OR puber*[tiab]) OR ("Adult"[Mesh] OR adult*[tiab] OR man[tiab] OR men[tiab] OR woman[tiab] OR women[tiab])

AND

(“Intervention program” [tiab] OR "Life Style"[Mesh] OR Lifestyle [tiab] OR “Life Style” [tiab] OR multi-component [tiab] OR multi-disciplinary [ tiab] OR interdisciplinary OR "health promotion*"[Title/Abstract] OR "evidence based program*"[Title/Abstract] OR "Health Promotion"[Mesh] OR “promotion of health” [tiab] OR "Healthy people program*”[tiab] OR “successful aging program*” [tiab] OR cross-disciplinary [tiab] OR “community-based intervention” [tiab] OR “health literacy” [tiab]) OR (("Diet"[Mesh] OR "diet*"[Title/Abstract] OR "diet therapy"[MeSH Terms] OR "eating"[MeSH Terms] OR dietar* [tiab] OR Dietetic*[tiab] OR Dieting [tiab] OR Dietic*[tiab] OR Dietit*[tiab] OR "dietary proteins"[MeSH Terms] OR "dietary supplements"[MeSH Terms] "eating"[Title/Abstract] OR "nutrition*"[Title/Abstract] OR "diet therapy" [Subheading] OR “protein supplementations” [tiab] OR protein* [tiab] ) AND ("resistance program*"[Title/Abstract] OR "resistance training"[Title/Abstract] OR "resistance training"[MeSH Terms] OR "exercise"[MeSH Terms] OR "exercise"[Title/Abstract] OR "physical activit*"[Title/Abstract] OR "strength training"[Title/Abstract] OR "Exercise Movement Techniques"[Mesh] OR "Exercise Therapy"[Mesh] OR "Sports"[Mesh] OR sport* [tiab] OR swimming [tiab] OR “weight-lifting”[tiab] OR “physical fitness”[tiab] OR running [tiab] OR "Running"[Mesh] OR Motor Activit*[tiab] OR Exercis*[tiab] OR Physical Exercis*[tiab] OR Isometric Exercis*[tiab] OR Aerobic Exercis*[tiab] OR aerobic* [tiab] OR training[tiab] OR stretching[tiab] OR Physical Condition*[tiab] OR Physical fitness[tiab] OR Physical endurance[tiab] OR movement therap*[tiab] OR fitness training[tiab] OR Weight-Bearing[tiab] OR jogging[tiab] OR walk*[tiab] OR bicycle[tiab] OR cycle[tiab] OR bicycling[tiab] OR cycling[tiab] OR rowing[tiab] OR swim*[tiab] OR ambulation[tiab] OR mobil*[tiab] OR pilates[tiab] OR yoga[tiab] ))

AND

("implementation science"[MeSH Terms] OR "implementation science"[Title/Abstract] OR "implement*"[Title/Abstract] OR "implementation process"[Title/Abstract] OR "health plan implementation"[MeSH Terms] OR "health plan implementation"[Title/Abstract] OR "adopt*"[Title/Abstract] OR "routin*"[Title/Abstract] OR "integrat*"[Title/Abstract] OR "uptake"[Title/Abstract] OR "implementation"[Title/Abstract] OR "diffusion of innovations"[Title/Abstract] OR "dissemination"[Title/Abstract] OR "knowledge to action*"[Title/Abstract] OR "knowledge transfer"[Title/Abstract] OR "knowledge translation"[Title/Abstract] OR "research to practice"[Title/Abstract] OR "research utilization"[Title/Abstract] OR "research utilisation"[Title/Abstract] OR "scale up"[Title/Abstract] OR "process evaluation"[Title/Abstract] OR “effectiveness research” [tiab] OR “translational research”[tiab])

AND

("primary care"[Title/Abstract] OR "Primary Health Care"[Mesh] OR "primary health care"[Title/Abstract] OR “general practi*” [tiab] OR “family practi*” [tiab] OR “primary care” [tiab] OR “ambulatory care” [tiab] OR "community"[Title/Abstract] OR "community-based"[Title/Abstract] OR "neighbourhood*"[Title/Abstract] OR "health center"[Title/Abstract] OR "health service*"[Title/Abstract] OR “health office*”[tiab] OR "residence"[Title/Abstract] OR "community medicine"[Title/Abstract] OR "community medicine"[MeSH Terms] OR "community health centers"[MeSH Terms] OR "community health services"[MeSH Terms] OR "primary health care"[MeSH Terms] OR "community health center*"[Title/Abstract] OR "community health services"[Title/Abstract] OR "community setting*"[Title/Abstract])
